# A novel l-isoleucine-4′-dioxygenase and l-isoleucine dihydroxylation cascade in *Pantoea ananatis*

**DOI:** 10.1002/mbo3.87

**Published:** 2013-04-02

**Authors:** Sergey V Smirnov, Pavel M Sokolov, Veronika A Kotlyarova, Natalya N Samsonova, Tomohiro Kodera, Masakazu Sugiyama, Takayoshi Torii, Makoto Hibi, Sakayu Shimizu, Kenzo Yokozeki, Jun Ogawa

**Affiliations:** 1 Ajinomoto-Genetika Research Institute1st Dorozhny pr. 1, Moscow, 113545, Russia; 2 Institute of Food Sciences and Technologies Food Product Division, Ajinomoto Co. Inc.Suzuki-cho, Kawasaki-ku, Kawasaki, 210-8681, Japan; 3 Research Institute for Bioscience Products and Fine Chemicals, Ajinomoto Co. Inc.Suzuki-cho, Kawasaki-ku, Kawasaki, 210-8681, Japan; 4 Industrial Microbiology, Graduate School of Agriculture, Kyoto UniversityKitashirakawa-oiwakecho, Sakyo-ku, Kyoto, 606-8502, Japan; 5 Division of Applied Life Sciences, Graduate School of Agriculture, Kyoto UniversityKitashirakawa-oiwakecho, Sakyo-ku, Kyoto, 606-8502, Japan

**Keywords:** 4-Hydroxyisoleucine, furanones, *Pantoea ananatis*, plant pathogenic bacteria, *Pseudomonas syringae*, quorum sensing

## Abstract

A unique operon structure has been identified in the genomes of several plant- and insect-associated bacteria. The distinguishing feature of this operon is the presence of tandem *hilA* and *hilB* genes encoding dioxygenases belonging to the PF13640 and PF10014 (BsmA) Pfam families, respectively. The genes encoding HilA and HilB from *Pantoea ananatis* AJ13355 were cloned and expressed in *Escherichia coli*. The culturing of *E. coli* cells expressing *hilA* (*E. coli*-HilA) or both *hilA* and *hilB* (*E. coli*-HilAB) in the presence of l-isoleucine resulted in the conversion of l-isoleucine into two novel biogenic compounds: l-4′-isoleucine and l-4,4′-dihydroxyisoleucine, respectively. In parallel, two novel enzymatic activities were detected in the crude cell lysates of the *E. coli*-HilA and *E. coli*-HilAB strains: l-isoleucine, 2-oxoglutarate: oxygen oxidoreductase (4′-hydroxylating) (HilA) and l-4′-hydroxyisoleucine, 2-oxoglutarate: oxygen oxidoreductase (4-hydroxylating) (HilB), respectively. Two hypotheses regarding the physiological significance of C-4(4′)-hydroxylation of l-isoleucine in bacteria are also discussed. According to first hypothesis, the l-isoleucine dihydroxylation cascade is involved in synthesis of dipeptide antibiotic in *P. ananatis*. Another unifying hypothesis is that the C-4(4′)-hydroxylation of l-isoleucine in bacteria could result in the synthesis of signal molecules belonging to two classes: 2(5H)-furanones and analogs of N-acyl homoserine lactone.

## Introduction

**Figure 1:**
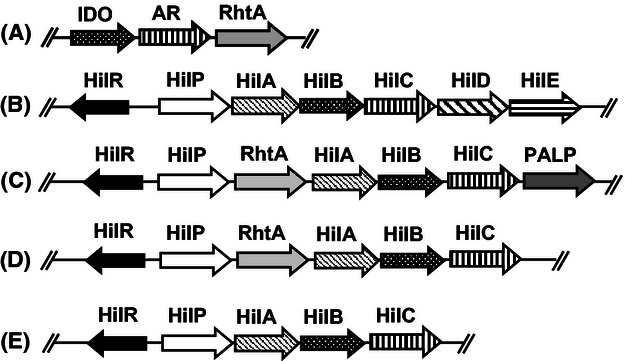
The structure of the Hil operons of plant- and insect-associated bacteria. The putative operon structures were deduced from the analysis of the following genomes. (A) *Bacillus thuringiensis* sp. 2e2 (Smirnov et al. 2012); (B) *Pantoea ananatis* AJ13355 (Hara et al. 2011), *P. ananatis* LMG 20103 (De Maayer et al. 2010), *P. ananatis* LMG 5342 (De Maayer et al. 2012), and *Rahnella aquatilis* CIP 78.65 (Martinez et al. 2012); (C) *Pseudomonas syringae* pv. *phaseolicola* 1448A (Joardar et al. 2005); (D) *Pseudomonas savastanoi* pv. *glycinea* (Qi et al. 2011); and (E) *Xenorhabdu*s *nematophila* ATCC 19061. The distinguishing feature of the Hil operon is the presence of a “core” of five genes: HilR – a putative LysR-type transcriptional regulator (Pfam family PF00126) (Schell 1993); HilP – a putative extracellular solute-binding protein (PF00497); HilA – a putative dioxygenase (PF13640); HilB – a dioxygenase (PF10014) that is a close homologue of l-isoleucine-4-dioxygenase (IDO) (Smirnov et al. 2012); and HilC – a short-chain dehydrogenase (PF00106) that is a close homologue of 2-amino-3-methyl-4-ketopentanoic acid (AMKP) reductase (AR). Other genes included in the Hil operon are bacterium specific: RhtA – putative exporters belonging to the RhtA family (Livshits et al. 2003); HilD – putative ATP-dependent carboxylate-amine ligase (PF13535); HilE – putative major facilitator superfamily (MFS) transporter (PF07690); and PALP – putative pyridoxal-phosphate dependent enzyme (PF00291).The arrows corresponding to the genes are not drawn to scale to enable all operons to be fit into one figure. The arrows indicating homologous proteins have the same shading.

The C-4-hydroxylation of free l-amino acids is a recently discovered secondary metabolism phenomenon in bacteria. l-Isoleucine-4-dioxygenase (IDO; systematic name: l-isoleucine, 2-oxoglutarate: oxygen oxidoreductase [4′-hydroxylating]), a member of the Pfam PF10014 family, was first found in *Bacillus thuringiensis* (Kodera et al. 2009). Since its discovery, this enzyme has been thoroughly characterized (Hibi et al. 2011) and is now used for the biotechnological synthesis of 4-hydroxyisoleucine, a natural nonproteinogenic amino acid with insulinotropic biological activities (Smirnov et al. 2010). Subsequent investigations revealed the existence of a novel subfamily of bacterial dioxygenases belonging to the PF10014 (BsmA) family (Weber et al. 2010) that accept free l-amino acids as substrates in vivo. l-leucine, l-isoleucine, and l-threonine were found to be hydroxylated by the PF10014 members, producing 4-hydroxyleucine, 4-hydroxyisoleucine, and 4-hydroxythreonine, respectively (Smirnov et al. 2012). A detailed analysis of the genetic organization of PF10014 members revealed a unique operon structure (designated the Hil operon, from the **h**ydroxylation of **i**so**l**eucine) in the genomes of several gram-negative plant pathogens belonging to the *Pantoea* and *Pseudomonas* genera (Smirnov et al. 2012) (Fig. 1). The distinguishing feature of this operon is the presence of tandem *hilA* and *hilB* genes encoding putative dioxygenases belonging to the PF13640 and PF10014 Pfam families, respectively. Previously, we found that HilB from *Pantoea ananatis* AJ13355 also catalyzes the C-4-hydroxylation of l-isoleucine, but the *K*_M_ value deduced from the kinetic analysis of this reaction was much greater than that determined for IDO, suggesting that l-isoleucine is not a natural substrate of HilB (Smirnov et al. 2012). In contrast to IDO, HilB is coexpressed with a putative dioxygenase, HilA. Moreover, in some bacteria, there is translational coupling between HilA and HilB, suggesting that these enzymes form a complex that catalyzes two consecutive reactions and implying that the natural substrate of HilB is a product of the reaction catalyzed by HilA. In this study, we provided evidence supporting this hypothesis and characterized the enzymatic activities of HilA/HilB from *P. ananatis* AJ13355.

## Materials and Methods

### Bacterial strains, plasmids, and oligonucleotides

The bacterial strains, plasmids, and oligonucleotides used in this study are listed in Tables 1 and 2.

**Table 1:** Strains, plasmids, and their characteristics

Strain or plasmid Strains	Relevant characteristics	Source or reference
TG_1_	*Escherichia coli supE hsd*Δ5*thi* Δ(*lac-proAB*)F′ [*traD36 proAB*^+^*lacI*^q^*lacZΔM15*]	(Carter et al. 1985)
PAA	*Pantoea ananatis* AJ13355, a gram-negative acidophilic bacterium isolated from the soil of a tea plantation in Iwata-shi (Shizuoka, Japan)	(Hara et al. 2011)
TG-HilA	*E. coli* TG_1_ strain harboring the pETAC-HilA^PAA^ plasmid	This study
TG-HilAB	*E. coli* TG_1_ strain harboring the pETAC-HilAB^PAA^ plasmid	
TG-C	*E. coli* TG_1_ strain harboring pUC19	
Plasmids		
pUC19	Ap^r^	(Vieira and Messing 1982)
pETAC-ilvA	Ap^r^. The plasmid was constructed by replacing the *Bgl*II-*Xba*I fragment of pET22b(+) (Novagen) with a synthetic *Bgl*II-*Xba*I fragment containing the P_*tac*_ promoter, followed by the cloning of the *E. coli ilvA* gene using the *Nde*I-*BamH*I sites.	Ajinomoto-Genetika Research Institute collection
pETAC-HilA	The hypothetical HilA^PAA^ protein (PAJ_3036; GenBank: BAK13116.1) from *P. ananatis* AJ13355 was expressed under the control of the P_*tac*_ promoter on a multicopy plasmid vector.	This study
pETAC-HilAB	The hypothetical HilA^PAA^ (PAJ_3036; GenBank: BAK13116.1) and HilB^PAA^ proteins (PAJ_3037; GenBank: BAK13117.1) from *P. ananatis* AJ13355 were expressed under the control of the P_*tac*_ promoter on a multicopy plasmid vector.	

**Table 2:** Oligonucleotides used in this study

Primer	Sequence (5′–3′)^1^
svs191	CGACTCTAGAGGATCCTTAAGAAGGAGATATACCATGACTGATTTACTGACCTTAGAACCTACGCAAAC
svs191^*^	GCTGGAGCTCTTATGTCCAGTAAACCACCTTACGG
svs190	GAATCGAGCTCAAGCTTCAGACCGGACATGCTGTCTTCTC
svs205	CGACTCTAGAGGATCCTTAAGAAGGAGATATACCATGCAGTCTGCAGCTTTGTTGGCGGTCTCAGGC
svs212	ATGCGAATTCGAGCTCTTATGTCCAGTAAATAACTTTACTACCTGA
svs206	CGGCCAGTGAATTCGAGCTCTTATCGGCTCCTCTGTTGAAACGTCACCAA

1 The specific restriction sites created (*Xba*I and *Sac*I) are underlined.

### Cloning of the *hilA* and *hilB* genes from *P. ananatis* AJ13355

To construct the pETAC-HilA and pETAC-HilAB plasmids, the following DNA fragments were amplified using the chromosomal DNA of the *P. ananatis* AJ13355 strain as a template: (1) a 935-bp fragment, “HilA,” using the primers svs191 and svs191*; and (2) a 1732-bp fragment, “HilAB,” using the primers svs191 and svs190. The resulting fragments were digested with *Xba*I and *Sac*I and then ligated into a pETAC-ilvA/*Xba*I-*Sac*I vector to yield the target plasmids.

### Low-scale biotransformation of l-isoleucine into l-4′-hydroxyisoleucine (4′-HIL) and l-4,4′-dihydroxyisoleucine (4,4′-DIHIL)

All constructed recombinant plasmids harboring the *hilA*/*hilAB* genes and pUC19 as a “control” plasmid were introduced into the *Escherichia coli* TG_1_ strain. The resulting plasmid strains were cultivated in 2 mL of TI medium (50 mmol/L KH_2_PO_4_ [pH 7, adjusted with KOH], 20 mmol/L NH_4_Cl, 2 mmol/L MgSO_4_, 1 mmol/L IPTG (isopropyl β-d-1-thiogalactopyranoside), 3 g/L l-isoleucine, 200 mg/L ampicillin, 10% [v/v] LB-broth, 10 g/L glucose, 1.25 g/100 mL chalk) at 32°C for 48 h in 20 mL test tubes with vigorous shaking. The final culture broth was analyzed using TLC (thin layer chromatography) and HPLC (high-performance liquid chromatography) analyses.

### Large-scale preparation and purification of 4′-HIL

The TG-HilA strain was cultivated in 750 mL flask containing 50 mL of LB broth at 37°C until *A*_540_ = 1 was reached. HilA expression was induced by adding IPTG to a final concentration of 1 mmol/L. Then, the induced cells were cultivated at 34°C for approximately 2.5 h. The obtained “active” biomass was harvested by centrifugation and resuspended in 5 mL of reaction buffer (50 mmol/L KH_2_PO_4_ pH 7, adjusted with NaOH; 20 mmol/L NH_4_Cl; 2 mmol/L MgSO_4_; 1.25 g/100 mL chalk; 1 mmol/L IPTG; 100 mmol/L l-isoleucine; and 100 mmol/L α-ketoglutarate). The resulting suspension was cultivated at 32°C for 48 h with vigorous shaking. Then, the cells were precipitated by centrifugation, and the resulting culture broth was filtered using a 0.45 μm CHROMAFIL Xtra CA-45/25 syringe filter and applied to a DOWEX 50WX2 column (1.3 × 16 cm) equilibrated with 30 μmol/L ammonia solution. An isocratic elution was performed using the same ammonia solution at a 1.5 mL/min elution rate, and 3 mL elution fractions were collected. Fractions containing 4′-HIL were pooled, lyophilized, and stored at −20°C until use.

### Large-scale preparation and purification of l-4,4′-DIHIL

The TG-HilAB strain was cultivated in LB medium (2 × 100 mL in 0.75 L flasks) at 37°C until OD_555_ = 1 was reached. The induction of HilA^PAA^ and HilB^PAA^ synthesis was carried out by IPTG adding to final concentration of 1 mmol/L. Then, the culture was incubated at 34°C for approximately 2.5 h. The cells were harvested and resuspended in 5 mL of reaction buffer RB (50 mmol/L KH_2_PO_4_ pH 7, adjusted with NaOH; 20 mmol/L NH_4_Cl; 2 mmol/L MgSO_4_; 100 mmol/L l-isoleucine; 150 mmol/L glycerol; and 100 mmol/L α-ketoglutarate). The resulting suspension was cultivated at 32°C for 16 h with vigorous shaking. Then, the following procedure was repeated twice: a 2.5 mL aliquot of culture broth was passed through a 0.45 μm CHROMAFIL Xtra CA-45/25 syringe filter and then applied to a DOWEX 50WX2 column (0.8 × 16 cm) equilibrated with 30 μmol/L ammonia solution. An isocratic elution was performed using the same ammonia solution at a 1.5 mL/min elution rate, and 3 mL elution fractions were collected. Fractions containing l-4,4′-dihydroxyisoleucine were pooled, lyophilized, and stored at −20°C until use.

### Analytical methods

l-Isoleucine hydroxylation was monitored by TLC analysis (n-BuOH:acetic acid: water = 12:3:5) using ninhydrin.

A high-pressure chromatography system with an 1100 series spectrofluorimeter (Agilent, Santa Clara, CA) was used for the HPLC analysis of the hydroxylation products. The detection used an excitation wavelength of 250 nm and an emission wavelength range of 320–560 nm. The amino acid derivative formation and separation were performed according to the manufacturer's recommendations (Waters). Separation by the AccQ-tag method was performed using an XBridge C18 column (2.1 × 150 mm, 5 μm) at 30°C with a flow rate of 0.3 mL/min. The mobile phases were AccQ-tag eluent A (eluent A) and acetonitrile (eluent B), and the flow rate of the eluent was 0.3 mL/min. The eluent gradients were 10–25% (v/v) B over 0–20 min, 60% (v/v) B for 20.1–23 min, and 10% (v/v) for 23.1–30 min.

LC/ESI-MS and ^1^H(^13^C)-NMR analyses of purified 4′-HIL and 4,4′-DIHIL were performed as was described in (Hibi et al. 2011).

### Purification of HilA

Cells of the TG-HilA strain from an LB-agar plate were inoculated into 1 L of LB broth (5 × 200 mL in 750 mL flasks) supplemented with Ap (100 mg/L), and the cultures were grown at 34°C until OD540 = 1 was reached. Then, protein expression was induced by adding IPTG (1 mmol/L) and incubating the culture for an additional 2 h. Then, the cells were harvested and stored at −70°C until use.

*Step 1*. The cells were thawed and resuspended in 40 mL of buffer A (50 mmol/L Tris pH 7, 50 mmol/L NaCl, 1 mmol/L DTT, 1 mmol/L EDTA). The cells were disrupted by two passages through a French pressure cell (maximum pressure, 2000 Psi) followed by centrifugation (14 000 g, 4°C, 20 min) to remove the cell debris.
*Step 2*. Forty milliliters of the protein preparation obtained from Step 1 was applied to a DEAE-Sepharose-Fast Flow (Sigma) column (1.6 × 25 cm) equilibrated with buffer A. The unbound (flow-through) fraction was collected.
*Step 3*. The protein obtained in Step 2 was precipitated by adding (NH_4_)_2_SO_4_ to 40% saturation and then resuspended in 2 mL of buffer A.
*Step 4*. One milliliter of the protein preparation obtained from Step 3 was applied to a Superdex 200 HR 10/30A column equilibrated with buffer A. An isocratic elution was performed at a flow rate of 0.5 mL/min. Each 1-mL fraction was collected. Active fractions were pooled and stored at −70°C until use.

### Purification of HilB

We performed expression and purification of his_6_-tagged HilB protein as was described previously in (Smirnov et al. 2012).

### Determination of dioxygenase activity

The reaction mixture (50 μL) contained 100 mmol/L HEPES (pH 7 adjusted with KOH), 5 mmol/L Ile, 10 mmol/L α-ketoglutarate, 5 mmol/L l-ascorbate, 5 mmol/L FeSO_4,_ and an aliquot of the protein preparation (20–50 μg of total protein). The purified l-4′-hydroxyisoleucine (≍5 mmol/L, see above) was used in reaction with purified HilB instead l-isoleucine. The reaction was carried out at 34°C for 1 h with vigorous shaking. The reaction products (l-4′-hydroxyisoleucine and l-4,4-dihydoxyisoleucine) were detected using TLC and/or HPLC analysis.

## Results

Because HilA and HilB are dioxygenases, it could be hypothesized that these enzymes catalyze the consecutive hydroxylation of an unknown substrate. Previously, we found that the substrate specificity profile of HilB for free amino acids is similar to that of IDO (for which l-isoleucine is a natural substrate) (Smirnov et al. 2012). Both enzymes catalyze the oxidation of l-methionine and the C-4-hydroxylation of l-leucine and l-isoleucine but exhibit different kinetic parameters for these reactions. The *K*_M_ and *V*_max_ values of both enzymes were similar for l-leucine but were very different for l-isoleucine and l-methionine. These data suggested that HilB could catalyze the C-4-hydroxylation of an unknown derivative of l-isoleucine. We hypothesized that the unknown compound is l-isoleucine hydroxylated by HilA at another C-position.

**Figure 2:**
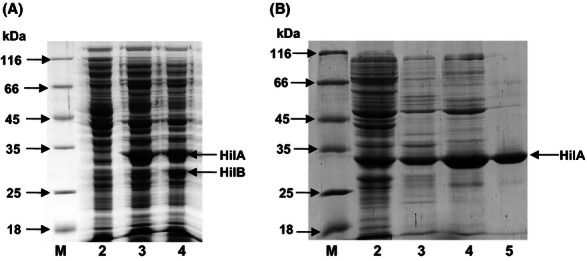
Expression and purification of HilA and HilB dioxygenases. (A) SDS-PAGE of total proteins from crude cell lysates of TG_1_ (lane 2), TG-HilA (lane 3), and TG-HilAB (lane 4) strains. All strains were cultivated in 20 mL test tube containing 5 mL of LB broth at 37°C until A_540_ = 1 was reached. HilA and HilB expression was induced by adding IPTG (isopropyl β-d-1-thiogalactopyranoside) to a final concentration of 1 mmol/L. Then, the induced cells were cultivated at 32°C for approximately 1 h. The obtained biomass was harvested by centrifugation, resuspended in 0.5 mL of 20 mmol/L Trsi-HCl pH8.0 buffer, and subjected to sonication treatment. Cell debris was removed by centrifugation and aliquot of resulting protein preparation (10 μg of total protein) was subjected to SDS-PAGE analysis. (B) SDS-PAGE analysis of protein preparations collected at the following steps of the HilA purification: lane 2 – crude cell lysate of the TG-HilA strain, lane 3 – anion exchange chromatography (flow-through fraction), lane 4 – ammonium sulfate fractionation, lane 5 – gel-filtration chromatography (pooled active fractions). M, molecular weight marker.

**Figure 3:**
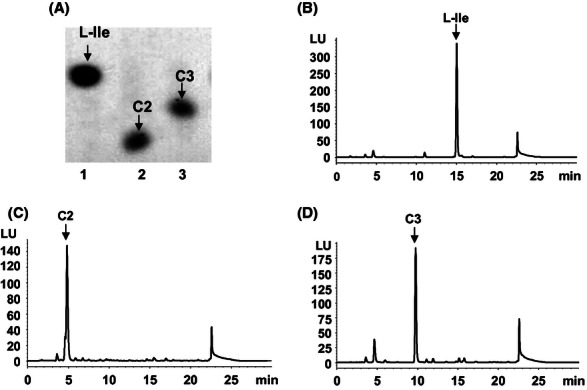
Detection of the novel hydroxylated derivatives of l-isoleucine produced by HilA/HilAB. (A) TLC analysis of the culture broths of the TG-C (lane 1), TG-HilAB (lane 2), and TG-HilA (lane 3) strains after cultivation in the presence of l-isoleucine. (B, C, D) HPLC analysis of the same culture broths, respectively. TLC spots and HPLC peaks corresponding to l-isoleucine (l-Ile), 4′-hydroxyisoleucine (C3), and 4,4′-hydroxyisoleucine (C2) are marked by arrows.

**Figure 4:**
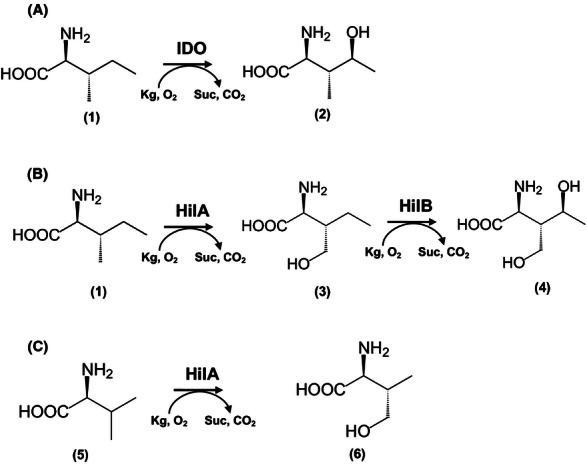
C-4(4′)-hydroxylation of l-isoleucine in bacteria. (A) The hydroxylation of l-isoleucine (1) by IDO from *Bacillus thuringiensis* results in the formation of 4-HIL (2). (B) The consecutive dihydroxylation of l-isoleucine catalyzed by HilAB results in the synthesis of the novel compounds 4′-hydroxyisoleucine (3) and 4,4′-dihydroxyisoleucine (4)., (C) The C-4 hydroxylation of l-valine (5) forms l-4-hydroxyvaline (6).

To test this hypothesis, we first investigated the in vivo activity of HilA with l-isoleucine. For this purpose, we cloned the *hilA* gene from *P. ananatis* AJ13355 into a multicopy vector under the transcriptional control of the strong P_*tac*_ promoter. The constructed plasmid was introduced into *E. coli* TG_1_ cells and the expression of HilA protein was confirmed by SDS-PAGE analysis of crude cell lysate of the resulting TG-HilA strain (Fig. 2A). Then, the TG-HilA strain was cultivated in synthetic medium supplemented with l-isoleucine. The TG_1_ strain harboring the pUC19 vector was also cultivated as a negative control under the same conditions. TLC and HPLC analyses of the resulting culture broth were then performed. These analyses showed that l-isoleucine was completely absent from the culture broth of the TG-HilA strain, and an unknown amino group-containing compound (C3) was detected (Fig. 3A and D). In the case of the TG-C strain, the l-isoleucine concentration was unchanged, and no new compounds were synthesized (Fig. 3B). These data explicitly suggest that l-isoleucine was converted into C3 due to the enzymatic activity of HilA. To further test this hypothesis, we investigated the l-isoleucine-dioxygenase activity of a crude cell lysate of TG-HilA. For this purpose, we performed a complete dioxygenase reaction and a set of control reactions, each lacking one essential component (l-isoleucine, α-ketoglutarate, l-ascorbate, or FeSO_4_). We found that the synthesis of C3 was strongly dependent on the presence of l-isoleucine, α-ketoglutarate, and FeSO_4_ (Table 3). We purified HilA from crude cell lysate of TG-HilA and investigated its dioxygenase activity with l-isoleucine in the same way. Resulting data were identical to those obtained with crude cell lysate of TG-HilA (Fig. 2B, Table 3). In addition, we confirmed dioxygenase activity of HilA by using a generic assay that uses succiny l-coenzyme A synthetase, pyruvate kinase, and lactate dehydrogenase to couple the formation of the product succinate to the conversion of reduced nicotinamide adenine dinucleotide to nicotinamide adenine dinucleotide (Luo et al. 2006). To determine the chemical structure of C3, it was purified and subjected to ^1^H-NMR, ^13^C-NMR, and ESI-MS analyses (Table 4). These data allowed us to identify C3 as l-4′-hydroxyisoleucine and HilA as an l-isoleucine, 2-oxoglutarate: oxygen oxidoreductase (4′-hydroxylating) (Fig. 4B). In addition to l-isoleucine, l-valine, and l-methionine were transformed by purified HilA (although the enzyme exhibited low activity with these substrates), resulting in the formation of 4-hydroxyvaline (identified using ^1^H NMR and ESI-MS analyses, data not shown) and presumably l-methionine sulfoxide, respectively (Fig. 4C).

**Table 3:** Synthesis of C2 and C3 by the dioxygenase reaction with different substrate compositions

	Reaction^3^				
	
Enzyme	A	B	C	D	E
HilA^1^	C3	nd^5^	nd	C3	nd
HilAB^1^	C2, C3^4^	nd	nd	C2, C3	nd
HilA^2^	C3	nd	nd	C3	nd
HilB^2^	C2	nd	nd	C2	nd
HilA/HilB^2^	C2, C3^4^	nd	nd	C2, C3	nd

1 The crude cell lysates of TG-HilA and TG-HilAB were used for the in vitro dioxygenase assay.

2 The purified HilA and/or purified his_6_-tagged HilB protein were used for dioxygenase assay.

3 The reaction mixture contained the following: A, all components; B, C, D, and E, the same as A but without l-isoleucine (l-4′-hydroxyisoleucine in reactions with HilB), α-ketoglutarate, l-ascorbate, and FeSO_4_, respectively.

4 If equimolar concentrations of l-isoleucine and α-ketoglutarate were used in the HilAB activity assay, then the formation of both C3 (4′-HIL) and C2 (4,4′-DIHIL) was detected, whereas a twofold excess of α-ketoglutarate resulted in the complete conversion of l-isoleucine into C2.

5 Not detected.

**Table 4:** Identification of l-isoleucine hydroxylation products

	Spectrum		
		
Compound	^1^H-NMR	^13^C-NMR	ESI-MS m/z [M+H]
l-isoleucine^1^	–	δ 176.8 (C-1); 62.4 (C-2); 38.6 (C-3); 27.1(C-4′); 17.3 (C-4); 13.7 (C-5)	132
4′-HIL (C3)	δ 0.97(3H, *t*, *J* = 7.5 Hz)<δ>, 1.36–1.43 (2H, m) <γ>, 2.05–2.15 (1H, m)<β>, 3.71(1H, dd, *J* = 11.6, 6.8 Hz)<A1>, 3.81 (1H, dd, *J* = 11.6, 4.2 Hz), A2>, 3.94 (1H, d, *J* = 2.8 Hz)<α>	δ 176.9 (C-1); 64.8 (C-2); 60.1(C-4′); 45.05 (C-3); 21.4 (C-4); 14.0 (C-5)	148
4,4′-DIHIL (C2)	δ 1.30(3H, d, *J* = 6.4 Hz)<δ>, 2.19–2.24 (1H, m) <β>, 3.73 (1H, dd, *J* = 11.5, 7.6 Hz)<A1>, 3.82(1H, dd, *J* = 11.5, 4.8 Hz)<A1>, 3.99–4.06 (1H, m)<γ>, 4.07 (1H, d, *J* = 2.7 Hz)<α>	δ 176.7 (C-1); 68.8 (C-4); 64.1 (C-2); 57.5(C-4′); 50.1 (C-3); 24.0 (C-5)	164

1 The ^13^C-NMR spectrum of l-isoleucine was reproduced from HMDB (Wishart et al. 2009).

Previously, using the method described in (Smirnov et al. 2010), we found that *E. coli* cells expressing HilB convert l-isoleucine into 4-hydroxyisoleucine but that this conversion is much less effective than that using IDO (S. V. Smirnov, unpubl. data). Thus, when expressed without HilA, HilB acts as a minimally effective IDO (Fig. 4A). To determine how HilB functions in the presence of HilA in vivo, we cloned and expressed a tandem *hilA*-*hilB* construct in *E. coli* (Fig. 2B). The TG-HilAB strain was cultivated in the presence of l-isoleucine, as described above for the TG-HilA strain. The TLC and HPLC analyses of the resulting culture broth showed that l-isoleucine was completely absent from the culture broth of the TG-HilAB strain, and an unknown amino group-containing compound (C2) was detected (Fig. 3A and C). We identified C2 as 4,4′-DIHIL (Table 4, Fig. 4B). The α-ketoglutarate/Fe^2+^-dependent synthesis of l-4,4′-DIHIL from l-isoleucine was detected in the crude cell lysate of the TG-HilAB strain (Table 3). If equimolar concentrations of l-isoleucine and α-ketoglutarate were used in the activity assay, then the formation of both 4′-HIL and 4,4′-DIHIL was detected, whereas a twofold excess of α-ketoglutarate resulted in the complete conversion of l-isoleucine into l-4,4′-DIHIL. The in vitro investigation of combined dioxygenase activity of purified HilA and HilB revealed the same results. Finally, the purified HilB exhibited an α-ketoglutarate/Fe^2+^-dependent l-4′-hydroxyisoleucine-4-dioxygenase activity (Table 3).

These data allowed us to identify HilB as an l-4′-hydroxyisoleucine, 2-oxoglutarate: oxygen oxidoreductase (4-hydroxylating) (Fig. 4B). Thus, we can conclude that HilA and HilB catalyze the consecutive hydroxylation of l-isoleucine at the C-4′ and C-4 positions, respectively, to synthesize l-4′,4-dihydroxyisoleucine (Fig. 4B).

## Discussion

Many natural pharmaceutically active compounds with anti-cancer, antibiotic, and immunosuppressant activities are produced by microbial secondary metabolism (Demain 1999; Sithranga Boopathy and Kathiresan 2010; Vaishnav and Demain 2011). In this context, the newly discovered secondary metabolites 4′-HIL and 4,4′-DIHIL may also be used in pharmaceutical biotechnology. Indeed, both compounds are close “chemical homologues” of 4-HIL – a natural bioactive molecule that significantly decreases plasma triglyceride levels, total cholesterol, and fatty acid levels and increases glucose-induced insulin release in human and rat pancreatic islet cells in vitro (Jette et al. 2009). Thus, it would be very interesting to investigate the analogous biological activity of novel forms of hydroxylated l-isoleucine. If these molecules are used by the modern pharmaceutical industry in the future, their synthesis from l-isoleucine can be easily carried out using the method described in (Smirnov et al. 2010).

**Figure 5:**
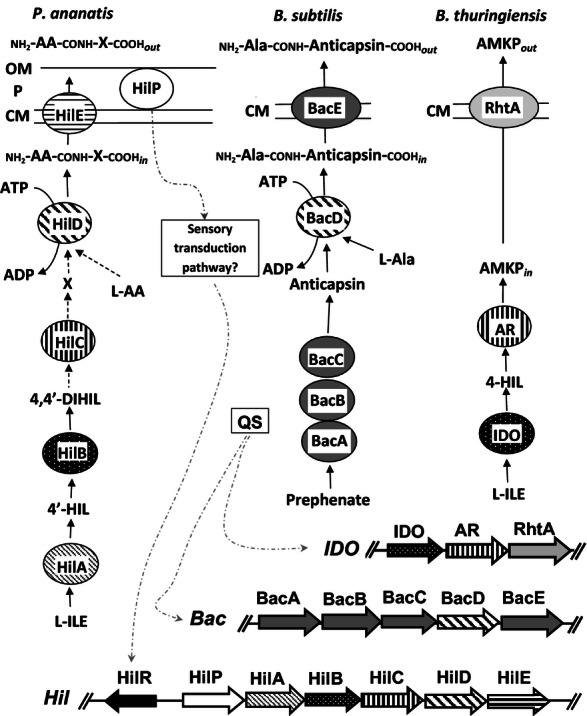
Comparative structural–functional analysis of the IDO, Bac, and Hil operons. In the insect pathogen *Bacillus thuringiensis*, the oxidation of 4-HIL catalyzed by 2-amino-3-methyl-4-ketopentanoic acid (AMKP) reductase (AR) results in the synthesis of an antimicrobial agent, AMKP, which is presumably then excreted by efflux pumps belonging to the RhtA exporter family (Ogawa et al. 2011). The genes encoding the hydroxylase, the reductase, and the exporter form an operon structure, IDO operon. Bacilysin consists of an l-alanine residue at the N terminus attached to a nonproteinogenic amino acid, l-anticapsin *via* amide (CONH) bond. l-anticapsin is synthesized from prephenate due to activities of BacABC enzymes and ligated with l-alanine by l-amino acid ligase BacD (Tabata et al. 2005). The resulting bacilysin is then exported from cell by BacE exporter. It was shown that the synthesis of this antibiotic is likely to be regulated by a quorum sensing (QS) pathway that is responsible for sporulation, competence development, and surfactant synthesis (Inaoka et al. 2003). By analogy, we can propose that the HilABC enzymes catalyze synthesis of l-isoleucine derivative that can be ligated with l-amino acid by the ATP-dependent carboxylate-amine ligase HilD and the resulting dipeptide antibiotic is then excreted from cell by the exporter HilE (Fig. 5). The putative QS-dependent expression of the Hil operon could be regulated by means of transcriptional regulator HilR and/or special sensory transduction pathway that could be initiated by binding of extracellular signal molecule with the extracellular solute-binding protein HilP. CM, cytoplasmic membrane; P, periplasm; OM, outer membrane; l-AA, l-amino acid.

Plant-associated bacteria (epiphytes and endophytes) serve as an important source of bioactive molecules. These bacteria produce numerous secondary metabolites that are involved in plant–bacterium and interbacterial interactions, including plant pathogenesis and quorum sensing/quenching-related processes (Strobel 2003; Gunatilaka 2006; Pimentel et al. 2011).It is notable that the Hil operon is found exclusively in plant and insect-associated bacteria. Our first insight into the physiological role for this operon is based on the noticeable structural–functional similarity between the Hill operon and the IDO and Bac operons from *Bacillus subtilis* (Fig. 5).

Abstracting from specific biochemical details, it can be argued that all three operons provide synthesis and excretion of biologically active substances, the effect of which is directed against environmental microbiota. As the toxic compounds are synthesized within the bacterial cell in each case, the special mechanism of self-detoxification is needed to prevent self-killing of bacteria. Thus, from the functional point of view, each operon contains two primary gene groups. First group consists of genes encoding proteins synthesizing toxic substance. Second group includes genes encoding proteins providing self-detoxification of cell.

2-amino-3-methyl-4-ketopentanoic acid (AMKP) synthesized by the *B. thuringiensis* is a “conditional” antibiotic that inhibits the growth of *Escherichia coli*, *Staphylococcus aureus*, *Sarcina lutea*, *B. subtilis*, and *Pseudomonas aeruginosa* growing only in the glucose minimal salts medium. No inhibitory activity was noted when the organisms were grown in a peptone-yeast extract medium, presumably because methionine, l-valine, l-leucine, and l-isoleucine present reversed the inhibition of the growth of the test organisms (Perlman et al. 1977). Thus, the active export of AMKP by RhtA seems to be enough for self-detoxification of *B. thuringiensis*.

On the contrary, anticapsin is a strong competitive inhibitor of house-keeping enzyme glucosamine synthase (GS). The irreversible inhibition of GS results in the lysis of bacterial or fungal cells. In this case, *B. subtilis* uses additional mechanism of “autodetoxification” – synthesis of dipeptide bacilysin in which the toxic anticapsin is “hidden” by binding with l-alanine. The antimicrobial activity of anticapsin is then initiated by reuptake and proteolysis of bacilysin by a peptidase to release anticapsin in an environmental target cell.

In the Hil operon, the expression of HilA and HilB is also coupled with the expression of an AR homologue (HilC), suggesting that oxidized 4,4′-DIHIL may be a novel antimicrobial agent. May be the “power” of this compound is comparable with that of anticapsin and may be that is why the Hil operon contains the BacD homologue, putative l-amino acid ligase HilD, thus providing the self-detoxification by mechanism used in *B. subtilis* (Fig. 5).

Because some “bearers” of the Hil operon (*Pantoea*, *Rahnella, Pseudomonas*) are plant epiphytes, further investigation of the Hil operon may be very useful for the construction of effective biological control agents with a broad activity spectrum against phytopathogenic bacteria (Chen et al. 2009).

**Figure 6:**
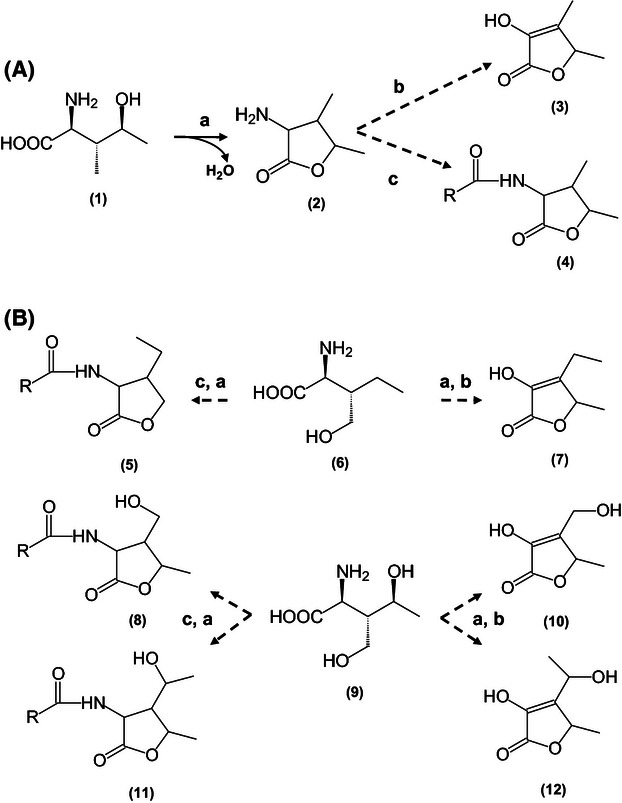
The C-4(4′)-hydroxylation of free l-isoleucine as a putative pathway for the synthesis of novel signaling molecules. (A) The lactonization (a) of 4-HIL followed by oxidative deamination (b) or amide bond formation (c) could result in the synthesis of 3-hydroxy-4,5-dimethyl-2(5H)-furanone (sotolon) (3) or *N*-acyl homoserine lactone analogs (4). (B) An analogous pathway may be proposed for the synthesis of the corresponding 2(5H)-furanones (7, 10, 12) and *N*-acyl homoserine lactone derivatives (5, 8, 11) from 4′-HIL (6) and 4,4′-dihydroxyisoleucine (9).

It is interesting to note that the C-4(4′)-hydroxylation of l-isoleucine (and other free l-amino acids) could, in theory, result in the synthesis of signal molecules belonging to two classes: 2(5H)-furanones and analogs of N-acyl homoserine lactone (AHL). Lactone formation followed by the Strecker reaction can directly yield 3-hydroxy-4,5-dimethyl-2(5H)-furanone (sotolon) from 4-HIL (Blank et al. 1996) (Fig. 6A). An analogous pathway may be proposed for the synthesis of the corresponding 2(5H)-furanones from all known 4-hydroxyamino acids, including 4′-HIL and 4,4′-dihydroxyisoleucine (Fig. 6B). It is well known that insects can transfer plant/insect pathogenic bacteria from one habitat to other (Nadarasah and Stavrinides 2011). Thus, we hypothesized that, due to the phenomenon of C-4(4′)-hydroxylation, plant-associated bacteria synthesize volatile, strong-smelling insect attractants to facilitate the spread of these bacteria.

The lactonization of C-4(4′)-hydroxylated free l-amino acids also enables the synthesis of AHL derivatives. This putative pathway could include lactonization and the formation of an amide bond between the carboxyl group of the “tail” (RCOOH) compound and the amino group of the “head” lactone molecule (Fig. 6). The resulting compounds could be used for the modulation of AHL and AI-2 quorum sensing pathways (Galloway et al. 2011). In this context, it is interesting to note that the ATP-dependent carboxylate-amine ligase HilD could be involved in the ligation of 4′-HIL or/and 4,4′-DIHIL with an unknown “tail” carboxylate.
